# Mineralization, anti-demineralization, and antibacterial effects of novel Bioactive Universal Bond with calcium salt monomers on dental caries

**DOI:** 10.3389/fdmed.2025.1633158

**Published:** 2025-09-15

**Authors:** Bayarchimeg Altankhishig, Yasuhiro Matsuda, Yaxin Rao, Takashi Saito

**Affiliations:** Division of Clinical Cariology and Endodontology, Department of Oral Rehabilitation, School of Dentistry, Health Sciences University of Hokkaido, Tobetsu, Japan

**Keywords:** CMET, Bioactive Universal Bond, mineralization, anti-demineralization, antibacterial effect, multifunctional material

## Abstract

This study investigated the mineralization, anti-demineralization, and antibacterial properties of a novel bioactive universal adhesive containing the calcium salt of 4-methacryloxyethyl trimellitate acid (CMET). Four adhesives were evaluated: Bioactive Universal Bond with CMET (BA), Clearfil Universal Bond Quick (CU), Prime & Bond Universal (PB), and Scotchbond Universal Plus Adhesive (SUB). To assess mineralization properties, BA specimens were immersed in simulated body fluid (SBF) at 37°C, in line with ISO 23317, and the induced crystals were analyzed using scanning electron microscopy and energy dispersive x-ray spectroscopy. Anti-demineralization was evaluated by applying each adhesive to polished bovine dentin, followed by a one-week pH cycling protocol. Transverse microradiography (TMR) was used to quantify the integrated mineral loss. The antibacterial activity was assessed using eluates prepared by immersing the cured specimens in distilled water for seven days, followed by serial dilutions (10%, 5%, 1%, and 0.5%). These eluates were incubated with *Streptococcus mutans*, *Actinomyces viscosus*, and *Lactobacillus casei* for 24 h at 37°C. Viable bacterial counts were determined using the quantitative polymerase chain reaction following propidium monoazide treatment. BA exhibited distinct mineralization in SBF, likely attributable to CMET, and was characterized by the formation of octacalcium-phosphate-like crystals. TMR analysis showed that BA significantly suppressed demineralization at the dentin–material interface in relation to the other adhesives. In antibacterial assays, 5% and 10% BA eluates markedly suppressed the growth of *S. mutans* and *A. viscosus,* whereas CU, PB, and SUB exhibited inhibitory effects at only 10% concentration for *S. mutans* and had no impact on *A. viscosus*. Notably, only the 10% BA eluate significantly inhibited *L. casei* growth. Despite the limitations of the *in vitro* experiments, these findings suggest that BA possesses multifunctional properties, supporting its potential as an effective adhesive system for the prevention and treatment of caries. Furthermore, its demonstrated bioactivity suggests promising applications across various biomedical fields, such as antibacterial coatings for medical devices, bone-regenerative scaffolds, and bioactive interfaces in tissue engineering and regenerative medicine.

## 1 Introduction

As a prevalent global health concern, dental caries is characterized by the demineralization of tooth structures due to the acidic byproducts of bacterial metabolism (1, 2). Maintaining a dynamic balance between demineralization and remineralization is crucial for preserving dental health (3). Therefore, effective management of both cariogenic bacteria and demineralized tooth substrates is essential for dental restoration and caries prevention (4).

For many years, research and development of adhesive materials for caries treatment have primarily focused on enhancing adhesive performance. However, contemporary adhesive dentistry has shifted attention toward the development of bioactive materials with remineralizing and antibacterial properties. These materials aim to prevent the recurrence of caries at the tooth–resin interface and the consequent failure of resin restorations (5, 6). Bioactive compounds and molecules have been incorporated into adhesive materials, including remineralizing agents such as hydroxyapatite, calcium silicates, bioactive glasses, and calcium phosphates, as well as antibacterial agents such as chlorhexidine, cetylpyridinium chloride, silver nanoparticles, and antimicrobial monomer 12-methacryloyloxydodecylpyridinium bromide (MDPB) (7–14). However, the destruction of the tooth structure caused by dental caries is a result of complex processes (1, 2). The development of multifunctional bioactive materials exhibiting remineralizing, antibacterial, and odontoblast-differentiating properties, while simultaneously maintaining favorable mechanical characteristics is crucial for preserving healthy tooth structures and promoting reconstruction and regeneration following carious lesions.

4-Methacryloxyethyl trimellitic acid (4-MET) is an acidic adhesive monomer widely used in dental materials. Its incorporation has been demonstrated to enhance adhesion to both enamel and dentin (15). Moreover, 4-MET is frequently combined with methyl methacrylate (MMA) in a formulation known as the 4-META/MMA-TBB adhesive (16). Previous studies have demonstrated that 4-MET forms ionic bonds with calcium in hydroxyapatite. Additionally, it induces lower dentin demineralization than other acidic monomers, allowing functional monomers to achieve strong chemical bonding with dentin and providing an effective seal against microleakage (15).

**Figure 1:**
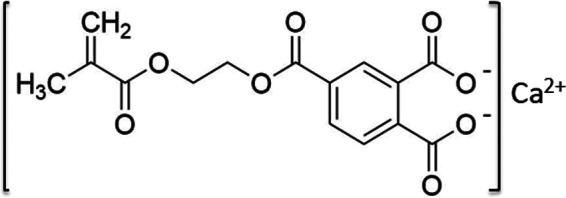
Chemical structure of CMET.

We developed a novel bioactive resin monomer, CMET, which is the calcium salt of 4-MET, as illustrated in Figure 1. The acidity of the molecule was modified by substituting calcium ions for hydrogen ions in the two carboxyl groups of 4-MET. This structural change is expected to enhance functionality, suggesting potential advantages for various dental applications and opening new directions for research (17).

Our previous studies demonstrated that CMET promotes mineral deposition in metastable solutions and exhibits antibacterial activity against several cariogenic bacterial species *in vitro* (17, 18). Furthermore, CMET facilitates the proliferation, odontoblastic differentiation, and mineralization of MDPC-23 cells *in vitro*, and has been shown to induce dentin regeneration when applied to exposed dental pulp tissue in a rat model simulating vital pulp therapy (19). Based on these findings, we defined CMET as a multifunctional bioactive monomer with the potential for dentin repair and regeneration, and we developed a novel universal adhesive incorporating it. In a recent study, we demonstrated that the CMET-containing universal adhesive exhibited favorable biocompatibility, including low cytotoxicity and the ability to promote odontoblastic differentiation *in vitro* (20). These findings indicate that the adhesive not only possesses bioactive functions but is also safe for biological applications.

This study aimed to evaluate the mineralization-inducing and anti-demineralization effects of a CMET-containing universal adhesive on bovine dentin as well as its antibacterial activity against three representative cariogenic bacterial species in comparison with commercially available universal adhesives.

## 2 Materials and methods

### 2.1 Mineral induction

To prepare cured specimens for use in the crystal-forming ability test, the solvent component (20% ± 2%) of the Bioactive Universal Bond containing CMET (BA, Sun Medical, Moriyama, Shiga, Japan) was volatilized and mixed with the BA brush in a ratio of two drops of bonding agent to one brush. The resulting mixture was poured into a Teflon mold (*φ*10 ± 1 mm × 2 ± 1 mm), placed on a glass plate covered with cellophane, and secured with a clip. Light curing was performed using a PenCure 2000 unit (J. Morita Corp., Kyoto, Japan) at an intensity of 1,000 mW/cm^2^. The specimen was irradiated for 20 s from both the top of the mold and the glass plate beneath it.

Following curing, the specimen was removed from the mold and immersed in 22 ml of simulated body fluid (SBF) prepared in accordance with ISO 23317 (21), contained in a 50 ml tube, and incubated at 37°C. During immersion, the surface that was not in contact with the cellophane (i.e., the observation surface) was oriented downward. After four weeks, the specimen was retrieved from the SBF, rinsed with purified water, and air-dried with the observation surface facing up at room temperature (25°C) without heating.

The crystals on the specimen surfaces were photographed through field-emission scanning electron microscopy (FE-SEM; JSM-6701F, JEOL Ltd., Tokyo, Japan). Elemental analysis of the crystals was conducted using scanning electron microscopy-energy dispersive x-ray spectroscopy (SEM/EDS; JSM-IT200, JEOL Ltd., Tokyo, Japan). Prior to the analysis, the specimens were sputter-coated with a thin layer of platinum. The SEM/EDS analysis was performed under the following conditions: an accelerating voltage of 15 kV and a working distance of approximately 10 mm. The elemental composition was determined by point analysis of the mineralized regions. The Ca/P molar ratio was calculated from three randomly selected areas per specimen. The induced crystals were characterized based on their morphologies and Ca/P ratios.

### 2.2 Automatic pH cycling

Freshly extracted noncarious bovine incisors were purchased from Hokkaido Livestock Corporation CO., LTD. (Obihiro, Japan). The bovine teeth were polished with a 1,000 grit silicon carbide paper to expose the dentin surface under running water. The teeth were then cut by a low-speed diamond saw (Isomet, Buehler, Lake Bluff, IL, USA) into longitudinal section widths of 150 µm to collect four single sections, and the cut surfaces were covered with sticky wax (Envista, Tokyo, Japan), except for the polished part.

**Figure 2:**
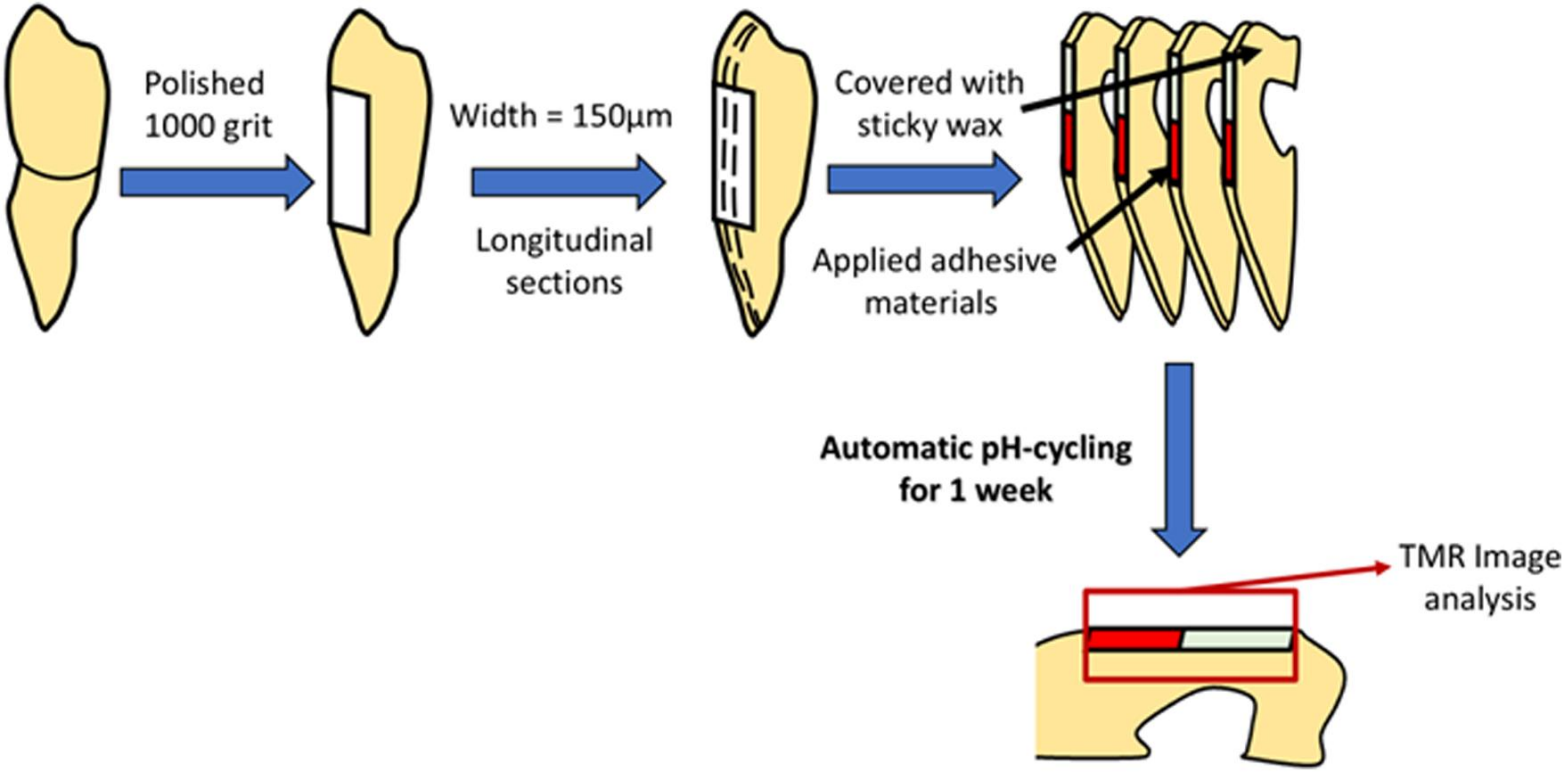
Experimental procedure for anti-demineralization analysis.

Four different materials were prepared for this study: BA, Clearfil Universal Bond Quick (CU) (Kuraray Noritake Dental, Tainai, Niigata, Japan), Prime & Bond universal (PB) (Dentsply Sirona, Charlotte, NC, USA), and Scotchbond Universal Plus Adhesive (SUB) (3M ESPE, St. Paul, MN, USA), as listed in Table 1. BA was mixed with a BA brush at a ratio of two drops of bonding agent to one brush and applied to the polished dentin surface. After volatilization of the solvent, light curing was performed using a PenCure 2000 unit at an intensity of 1,000 mW/cm^2^ for 5 s. The remaining three materials were partially applied to the polished surface in accordance with the manufacturer's instructions (Figure 2). All the specimens were placed in separate beakers for further analysis.

**Table 1:** Chemical formulations of the bonding agents.

Bonding agent	Chemical formulation	Manufacturer
Bioactive Universal Bond + brush (BA)	Bond: MDP, HEMA, ethanol, silane Brush: CMET, Chemical polymerization initiator	Sun Medical
Clearfil Universal Bond (CU)	Bis-GMA, Phosphate ester, MDP, HEMA, Hydrophilic amide monomer	Kuraray Noritake Dental
Prime & Bond Universal (PB)	Polyfunctional acrylate Phosphate ester monomer	Dentsply Sirona
Scotchbond Universal Plus Adhesive (SUB)	MDP, HEMA, Dimethacrylates, filler, Photoinitiator, ethanol, water, silane	3M ESPE

**Figure 3:**
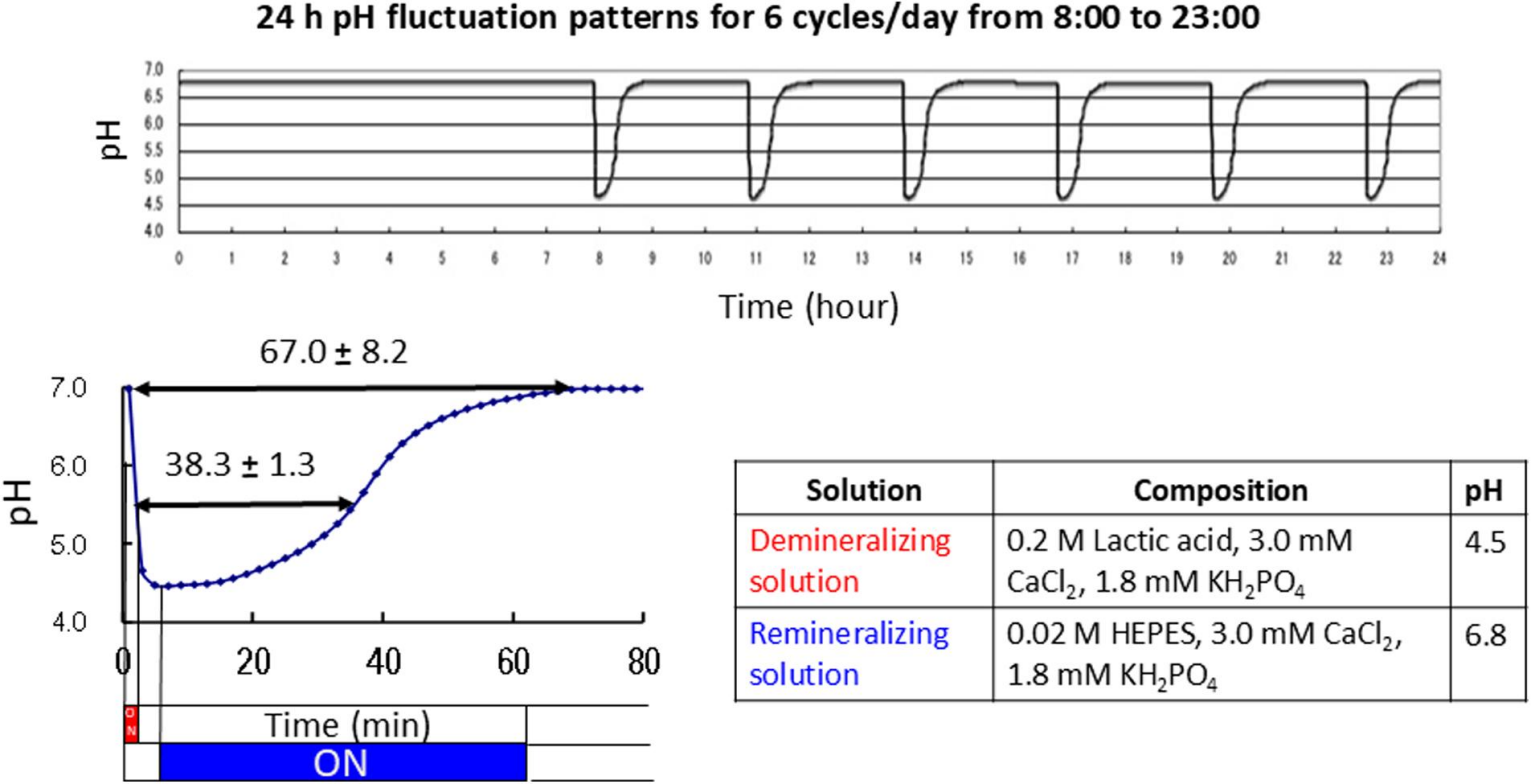
Daily pH cycling patterns and compositions of the demineralizing and remineralizing solutions.

An automatic pH cycling system was used according to the previous study conducted by Matsuda et al. (22). The demineralizing solution (pH 4.5), which consisted of 0.2 M Lactic acid, 3.0 mM CaCl_2_, and 1.8 mM KH_2_PO_4_, was injected into the beaker for 2 min. The remineralizing solution (pH 6.8), a mixture of 0.02 M 4-(2-hydroxyethyl)-1-piperazineethanesulfonic acid (HEPES), 3.0 mM CaCl_2_, and 1.8 mM KH_2_PO_4_, was gradually pumped in for 58 min. This process was repeated six times per day from 8:00 a.m. to 11:00 p.m. for one week (Figure 3).

### 2.3 Transverse microradiography imaging

Transverse microradiography (TMR) images were obtained to estimate the demineralization rate using a soft x-ray system (CSM-2; Softex Corp, Yokohama, Japan). Exposure was carried out for 20 min at 14 kV and 4 mA, with a focus-specimen distance of 44 mm, using an aluminum step wedge and high-resolution photo plates (HRP-SN-2, Konica Minolta, Tokyo, Japan). The digitization was performed using a microscope camera (PCM-500, As One, Tokyo, Japan) connected to an optical microscope (BX50, Olympus, Tokyo, Japan). Digitalized images before and after the pH cycling were superimposed using the image editing software (Adobe Photoshop Elements); furthermore, the area from the edge of the materials, a width of 50 μm, toward the depth was extracted as the analysis area. The degree of darkening of the TMR images before and after the pH cycle was analyzed using a general-purpose image analysis software (Image J, Bethesda, MD, USA). The obtained blackness was used to calculate the mineral profiles of the samples using the analysis program (23), and the integrated mineral loss (ΔIML) (Vol% × µm) was calculated (*n* = 5).

### 2.4 Antibacterial effect test

The test was conducted using *Streptococcus mutans (S. mutans)* (JCM 5705), *Actinomyces viscosus (A. viscosus)* (JCM1134), and *Lactobacillus casei (L. casei)* (JCM8353) strains. Each bacterial streak was spread on a brain heart infusion (BHI; Difco, Sparks, MD, USA) agar plate and incubated anaerobically at 37°C for 48 h. A single colony was picked from the plate, suspended in 10 ml of BHI broth, and incubated at 37°C for 24 h. Suspensions were centrifuged (40 rpm, 15 min at 4°C) and washed with phosphate-buffered saline (PBS; Thermo Fisher Scientific, Rockford, IL, USA) several times, and bacterial (OD1) absorbance was measured at a wavelength of 600 nm.

Four different one-step adhesive bonds were used to create hardened discs in a silicone mold. The discs were immersed in 20 ml of distilled water and incubated in a 37°C water bath for one week. The supernatant of each adhesive was collected and diluted to 10, 5, 1, and 0, 5%. The samples were then aliquoted with bacterial suspension in 96-well plates and incubated at 37°C for 24 h. After the incubation, all the samples were treated with propidium monoazide (PMA; Biotium, Fremont, CA, USA) and tested using quantitative polymerase chain reaction (qPCR, LightCycler96, Roche, Basel, Switzerland) (*n* = 5).

### 2.5 Statistical analyses

All results are expressed as mean ± standard deviation. Statistical analysis was performed using one-way analysis of variance (ANOVA), followed by Tukey's *post hoc* test. Statistical significance was set at *p* < 0.05. Prior to conducting ANOVA, the assumptions of normality and homogeneity of variance were verified using the Shapiro–Wilk test and Levene's test, respectively.

## 3 Results

### 3.1 Mineral induction effect

**Figure 4:**
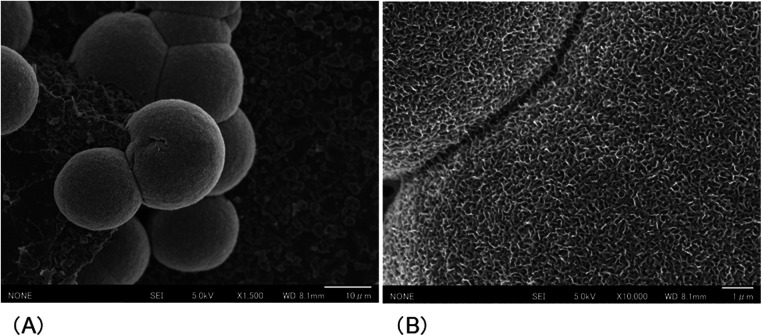
SEM images showing the morphology of the crystals formed on the surface of the cured BA disk after 21 d of immersion in SBF. **(A)** Spherical crystal aggregates **(B)** composed of thin, minute plate-like crystals were evident.

**Figure 5:**
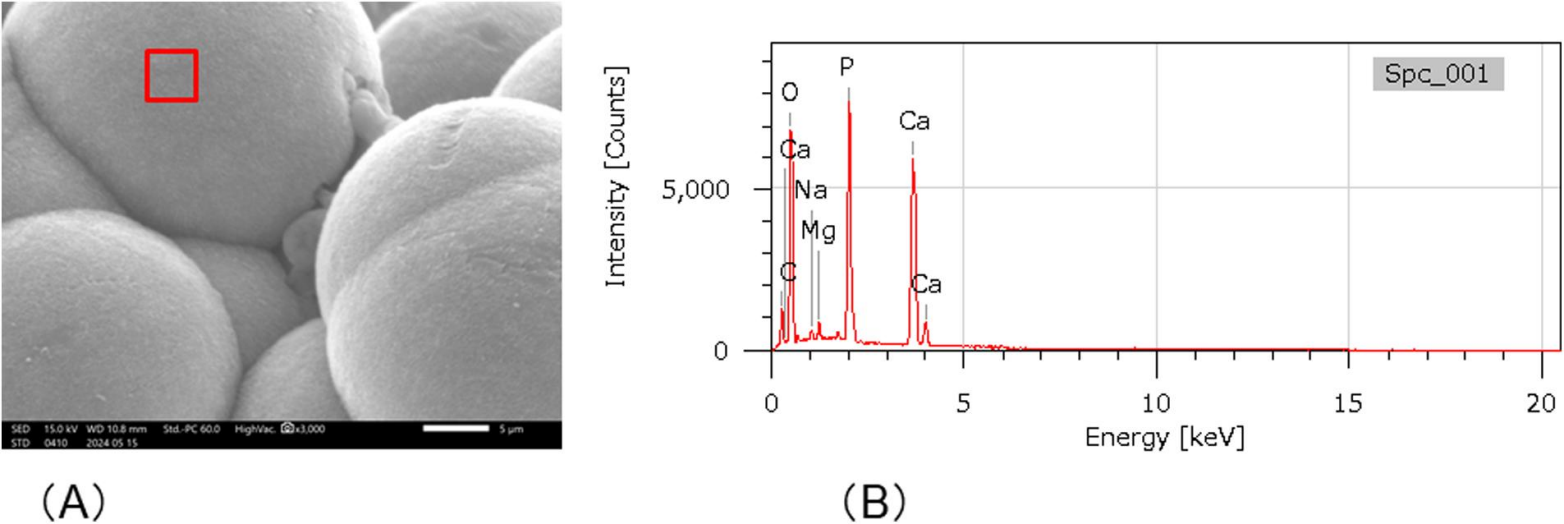
SEM-EDX analysis. **(A)** SEM images of crystal formation. Spectrum 1 shows the area of the SEM-EDS analysis. **(B)** SEM-EDX analysis of Spectrum 1 in **(A)**.

After the cured disk was immersed in the SBF for 21 d, it was dried, and the crystals deposited on the surface were observed through SEM. As a result, spherical crystal aggregations of *φ*8–40 µm were dotted over the entire surface of the cured disk. As the crystals grew, several aggregates were observed to merge, indicating a process in which small aggregations combined to form larger structures (Figure 4A). The spherical crystal aggregates were composed of minute plates (Figure 4B). Furthermore, the plate-like crystals were continuously connected, and the structure also formed a space of approximately 100 nm. EDS analysis showed that the Ca/P molar ratio of the crystals induced on the cured disk of the BA was 1.35 ± 0.13 (Figure 5; Table 2).

**Table 2:** Elemental compositions (atom %) of the specimen surface precipitates detected with EDS analysis.

Spectrum	C	O	F	Na	Mg	Si	P	Ca	Zr	Total
1	16.54	59.07	–	0.55	0.56	–	10.31	21.37	–	100
2	19.49	41.78	1.94	0.55	0.56	–	14.30	21.37	–	100
3	23.00	56.34	–	0.36	0.53	0.47	7.98	10.47	0.85	100

### 3.2 Anti-demineralization effect

**Figure 6:**
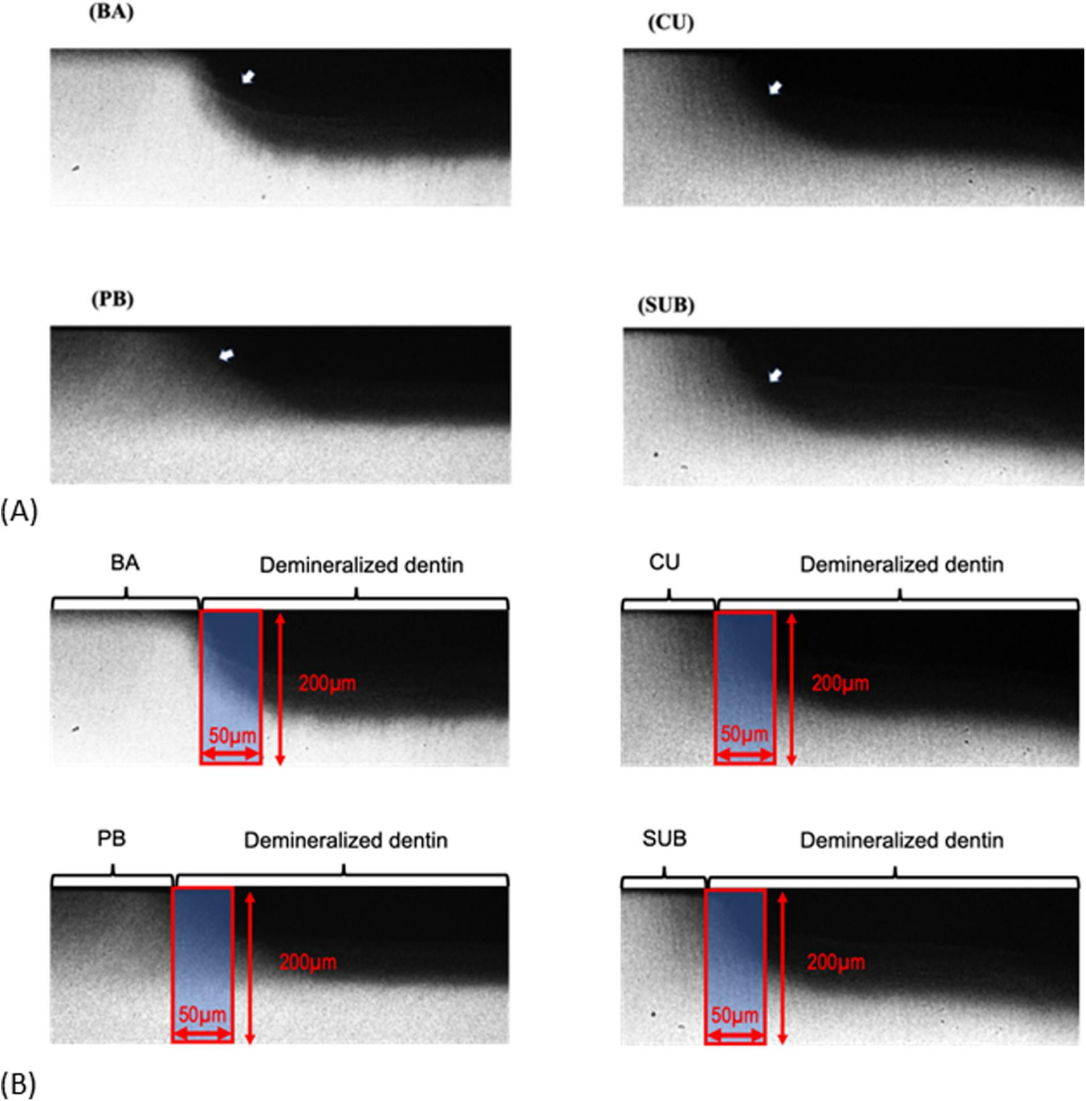
TMR image of the demineralized dentin exposed to various adhesives after 1 week of cariogenic pH cycling. **(A)** Dark areas represented by arrows indicate mineral loss. **(B)** Area (red square) used for calculating the mineral loss. BA, Bioactive Universal Bond; CU, Clearfil Universal Bond; PB, Prime & Bond Universal; SUB, Scotchbond Universal Plus Adhesive.

**Figure 7:**
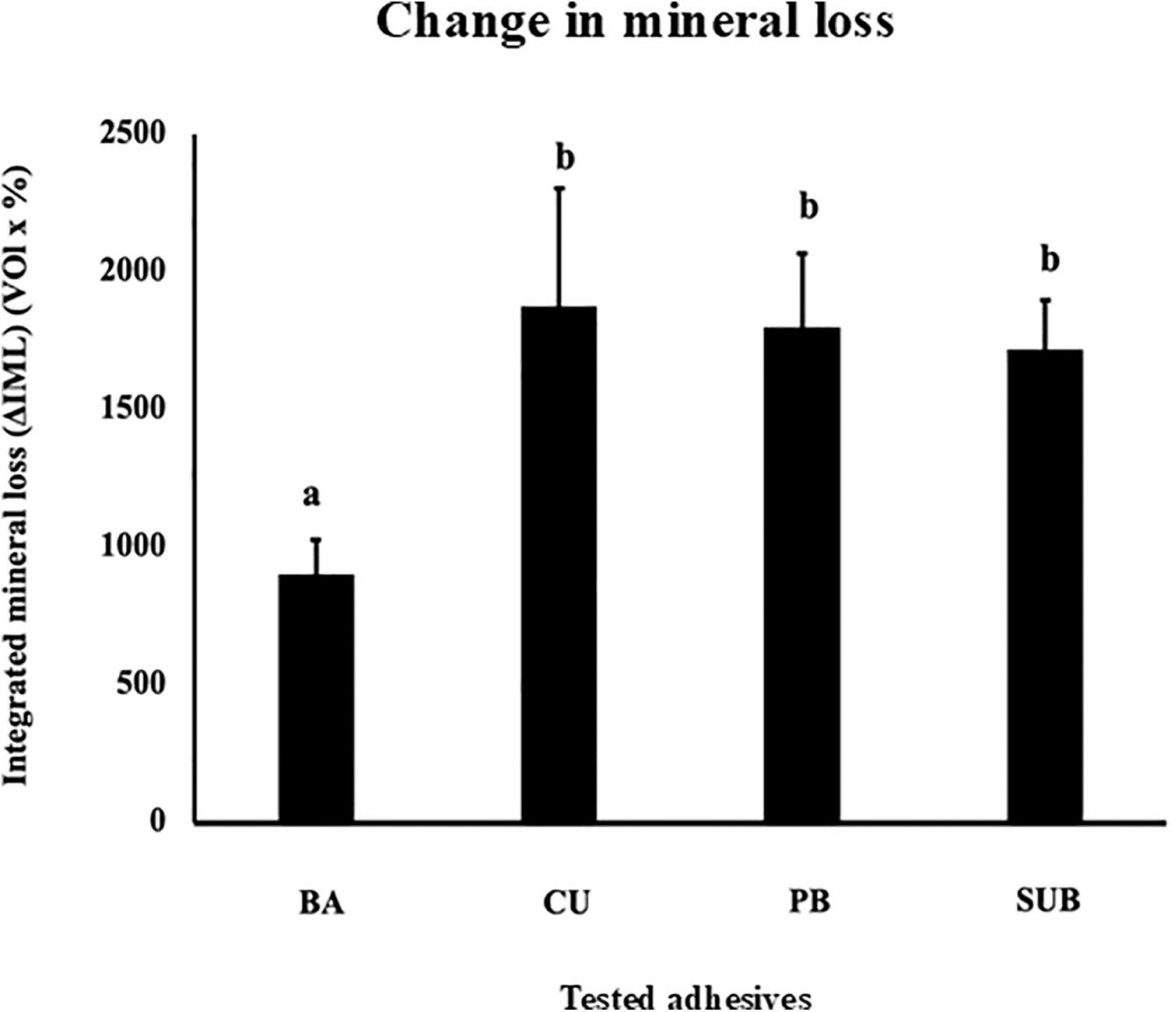
Change in the mineral loss (ΔIML) after 1 week of cariogenic pH cycling. Different letters indicate a significant difference in the mineral loss observed for the different adhesives (*n* = 5, one-way ANOVA and Tukey's tests, *p* < 0.05).

Figure 6A shows the TMR images of the dentin demineralization rates for the four different adhesive materials. To analyze the mineral loss from the edge of the applied material, an area with a width of 50 μm and depth of 200 μm was extracted (Figure 6B). The demineralization rate results showed that dentin with BA had significantly lower ΔIML than that with other three adhesives (*p* *<* 0.05) (Figure 7).

### 3.3 Antibacterial effect test

**Figure 8:**
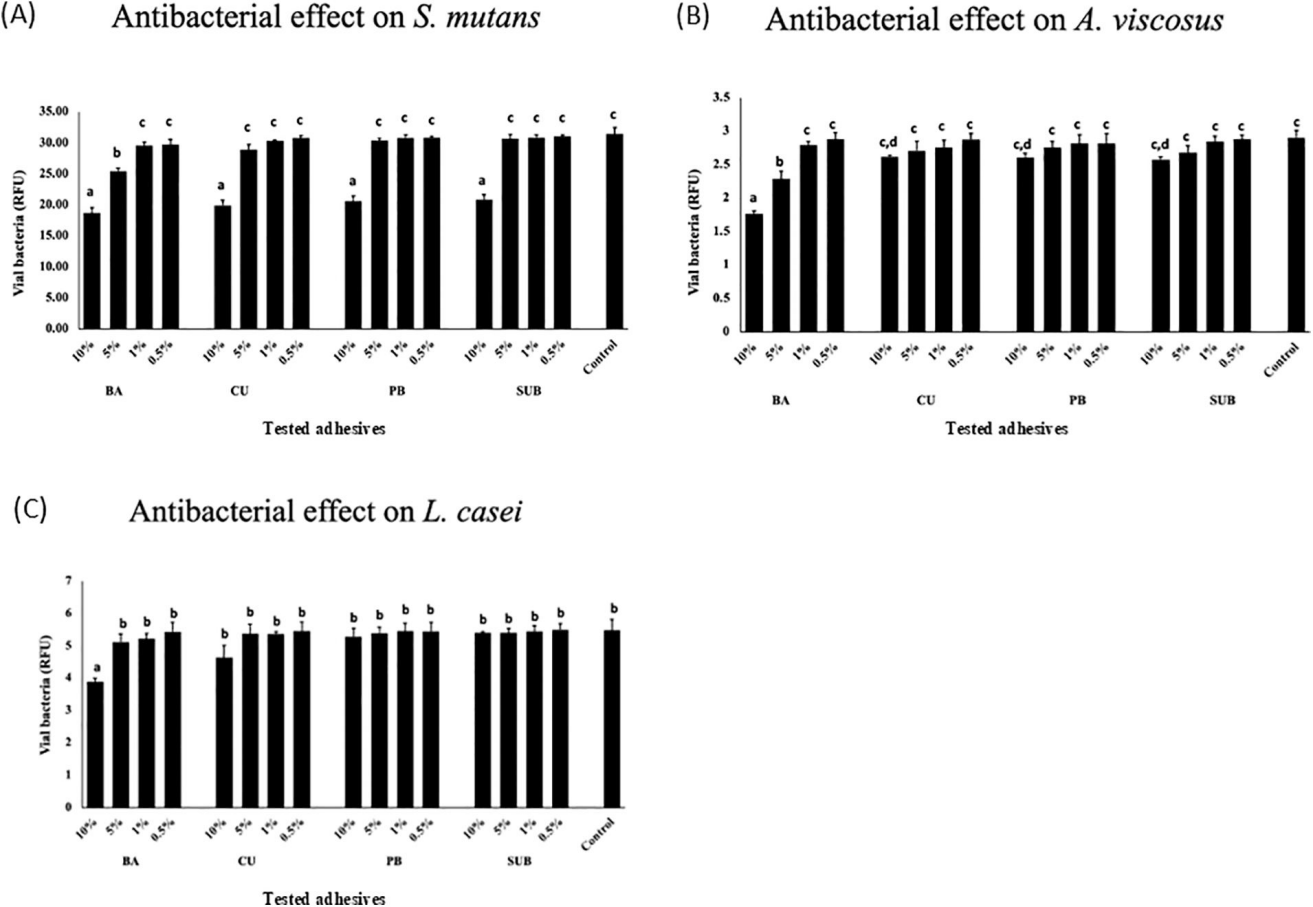
Antibacterial effects of the adhesives on *S. mutans* **(A)**, *A. viscosus*, **(B)** *and L. casei* **(C)** Different letters indicate a significant difference in the antibacterial effects induced by the different adhesives (*n* = 5, one-way ANOVA and Tukey's tests, *p* *<* 0.05).

The antibacterial efficacy of the adhesives was tested using the PMA-qPCR method. The results showed that both 10% and 5% BA significantly inhibited the growth of *S. mutans* in relation to the control group (*p* < 0.05). In contrast, among the CU, PB, and SUB groups, only 10% concentration demonstrated a statistically significant effect (Figure 8A). For *A. viscosus*, a similar pattern was observed: 10% and 5% BA significantly suppressed bacterial growth, whereas only 10% concentration of CU, PB, and SUB induced a comparable inhibitory effect (Figure 8B). In the case of *L. casei*, significant inhibition was observed exclusively for the 10% BA group (Figure 8C).

## 4 Discussion

CMET was initially developed because of its mineral-inducing potential (18). Subsequent studies have revealed that CMET also exhibits antibacterial activity and promotes odontoblastic differentiation (18, 19). On the basis of these findings, a novel bioactive adhesive material, BA Bond, was developed by incorporating CMET as a functional monomer.

Owing to its mineral-inducing ability, CMET facilitated the formation of mineral crystals on BA bond discs incubated in SBF, prepared according to ISO 23317. CMET has been shown to induce mineral deposition in a metastable calcium phosphate solution that did not spontaneously precipitate *in vitro*. It also significantly reduces the mineralization time of the decalcified dentin model when co-incubated. X-ray diffraction confirmed that the crystals formed by CMET were apatite (17).

Within the dentin matrix, phosphorylated proteins, such as phosphophoryn, play crucial roles in collagen mineralization and contribute to the structural integrity of dentin (24). Previous research has indicated that CMET-induced apatite formation mimics the process facilitated by phosphophoryn (17). In this study, the BA demonstrated results consistent with those reported previously for CMET. Microstructural analysis via SEM/EDS revealed that the crystals formed on the BA bond surface exhibited a plate-like morphology with a Ca/P ratio of 1.35, corresponding to that of octacalcium phosphate (OCP), a known precursor of hydroxyapatite (Ca/P molar ratio of 1.67) that transitions to its stable form under physiological conditions. Minor discrepancies in the EDS-derived compositions may result from instrumental variations or methodological differences (25). In this study, data were averaged from triplicate measurements per sample. These findings suggest that BA bonds promote the formation of OCP-like crystals *in vitro* owing to the ability of CMET to act as a nucleation agent for crystal formation.

This study also provides compelling evidence that CMET exerts significant anti-demineralization effects on dentin under pH cycling conditions. This model closely simulates the oral environment, which is characterized by alternating acidic and neutral periods conducive to remineralization (26). This study employed a newly designed automatic pH cycling system to simulate representative daily pH fluctuations (22, 23). The findings of this study are consistent with those reported previously, demonstrating the capacity of calcium- and phosphate-releasing materials, including bioactive glass and casein phosphopeptide-amorphous calcium phosphate (CPP-ACP), to enhance remineralization and dentinal tubules occlusion (27–29). Our results support the hypothesis that CMET not only protects the dentin matrix against acid-induced demineralization but also facilitates surface remineralization. The anti-demineralization mechanism of CMET is likely multifactorial. First, the release of calcium ions from CMET may shift the local ionic environment to favor remineralization, because supersaturation with calcium and phosphate ions is essential for hydroxyapatite reprecipitation. Second, the carboxylic acid groups in CMET can chelate calcium ions and serve as nucleation sites for the formation of apatite-like crystals. This interaction between CMET and hydroxyapatite may enhance dentin resistance to acid attack.

PMA-qPCR is a quantitative polymerase chain reaction technique that employs PMA to selectively inhibit DNA amplification from dead cells, thereby enabling the specific detection and quantification of DNA derived from viable cells (30). Using this method, the antibacterial properties of the BA were assessed using three representative cariogenic bacteria. *S. mutans*, a key pathogen in dental caries, can adhere to tooth surfaces, metabolize sugars, produce acids, and thrive in acidic environments. It forms glucan-based biofilms that facilitate colonization (31). *A. viscosus*, a gram-positive bacterium, adheres to tooth surfaces and ferments carbohydrates to produce acids. It also binds strongly to collagen on root surfaces and has been implicated in root caries development (32, 33). *L. casei*, frequently isolated from active carious lesions, does not efficiently adhere to tooth surfaces alone but can co-adhere with *S. mutans* (34). In this study, the BA bond exhibited potent concentration-dependent antibacterial effects. Previous studies have demonstrated the antibacterial efficacy of functional monomers, such as MDPB; however, these monomers lack mineralizing properties (35). In contrast, CMET offers multiple beneficial effects, as indicated by the results obtained in this study. The results suggest that the antimicrobial effects of CMET may derive from both its acidic monomer structure and calcium ion release, which can influence bacterial morphology and growth dynamics. To gain deeper insight into the mechanisms underlying the mineralization and antibacterial effects of CMET, future studies will aim to analyze calcium ion release kinetics, resin–dentin interactions at the molecular level, and changes in surface energy that may influence bacterial adhesion and mineral nucleation.

Although the findings reported here are promising, several limitations of the present study should be acknowledged. First, all experiments were conducted *in vitro*, which may not fully replicate the complex conditions of the oral cavity. Second, the small sample size for each experimental group may affect the statistical robustness of the results. Third, although the results confirm the antibacterial efficacy of BA against selected species, its full-spectrum antimicrobial profile and long-term durability remain to be investigated. Future studies should test the antibacterial efficacy of BA using dynamic oral biofilm models, long-term aging protocols, and clinical conditions to confirm its therapeutic potential in dentistry. In this study, the antibacterial activity of the adhesives was assessed using eluates prepared from cured specimens. However, we acknowledge that this method does not fully replicate the complex and dynamic conditions of the oral environment, where direct and prolonged contact between the material and biofilms occurs. Future studies should incorporate additional antibacterial assessment methods, such as direct contact tests, agar diffusion tests, or multispecies biofilm models, which more closely mimic clinical conditions and could provide a more comprehensive understanding of the antibacterial performance of the material *in situ*.

Despite these limitations, this study highlights the potential of CMET as a multifunctional component for adhesive systems. Other bioactive materials, such as bioactive glass, CPP-ACP, and calcium-silicate-based cements, have shown similar remineralizing effects (27–29, 36). However, only a few of these offer the combination of adhesion performance, remineralizing/anti-demineralization activities, and antibacterial functionalities within a single monomeric system. Recent studies have shown that the bond strength of CMET-based adhesives is comparable to that of conventional universal adhesives (37). Further studies are necessary to assess the mechanical and adhesive properties of the material. Comprehensive evaluation of bond strength, marginal integrity, and clinical performance will be essential to support the potential clinical application of this multifunctional adhesive. Regarding biosafety, a previous study by our group confirmed the biocompatibility of the CMET-containing universal adhesive, demonstrating minimal cytotoxicity and positive cellular responses *in vitro*, including odontoblast-like differentiation of pulp-derived cells (20). These findings support the material's potential for safe clinical use. However, additional studies involving long-term exposure and *in vivo* conditions are warranted to further validate its safety profile.

In this study, we developed a novel universal adhesive containing the bioactive monomer CMET, which exhibited multiple beneficial effects *in vitro*, including mineral induction, anti-demineralization, and antibacterial activity. These findings suggest that CMET may also enhance the performance of other dental materials such as liners, bases, and sealants.

## 5 Conclusions

This study demonstrated that the application of a novel bioactive universal adhesive containing CMET-BA to bovine dentin effectively induced mineralization and inhibited demineralization. Furthermore, BA exhibited pronounced antibacterial activity against *S. mutans*, *A. viscosus*, and *L. casei* in a concentration-dependent manner. These findings highlight the potential of BA as a promising candidate for the development of next-generation multifunctional dental materials with enhanced remineralization capacity, resistance to demineralization, and broad-spectrum antimicrobial properties. However, the present study is limited to *in vitro* experiments. Further investigations involving animal models and clinical trials are necessary to validate the results obtained in this study and to optimize CMET-based formulations for clinical applications. In addition to clinical dentistry, the multifunctional properties of CMET-based adhesives make them suitable for a broader range of biomedical and industrial applications such as antibacterial coatings for medical devices, mineralization-promoting scaffolds in bone tissue engineering, and bioactive interfaces in regenerative medicine.

## Data Availability

The raw data supporting the conclusions of this article will be made available by the authors, without undue reservation.
